# Using a Gratitude Intervention to Improve the Lives of Women With Breast Cancer: A Daily Diary Study

**DOI:** 10.3389/fpsyg.2019.01365

**Published:** 2019-06-12

**Authors:** Joanna Sztachańska, Izabela Krejtz, John B. Nezlek

**Affiliations:** ^1^SWPS University of Social Sciences and Humanities, Warsaw, Poland; ^2^SWPS University of Social Sciences and Humanities, Poznań, Poland; ^3^College of William and Mary, Williamsburg, VA, United States

**Keywords:** gratitude, breast cancer, diary study, well-being, multilevel modeling

## Abstract

Gratitude can be understood in two ways: as a state of being grateful for things and people, and as a disposition. Research suggests that focusing on reasons for being grateful promotes various aspects of well-being. The present study examined the effectiveness of a gratitude intervention for women with breast cancer. Each day for 2 weeks, 42 women with breast cancer described their psychological well-being, social support, and coping strategies. Women in the intervention condition reported the reasons why they felt grateful that day. Moreover, all participants took part in a pre-test session where trait measures were taken to control for dispositional differences. Listing the reasons for gratitude led to higher levels of daily psychological functioning, greater perceived support, and greater use of adaptive coping strategies. These results suggest that gratitude interventions may improve the lives of oncological patients.

## Introduction

The most prevalent type of cancer among women is breast cancer, which represents 25% of cancers in women with an estimated number of over 1.7 million new diagnoses each year (World Cancer Research Fund International, 2012). Moreover, advances in cancer treatment have led to “survivability rates” that can reach almost 90% (American Cancer Society, 2016). This means that although survivability rates can vary widely as a function of the location and type of cancer and the stage when it is diagnosed, the trend is such that many women are living with the disease for longer periods of time than in the past. In this sense, breast cancer is being transformed from a fatal illness into a chronic condition.

Although modern science has found ways to treat the physical disease of breast cancer, the negative psychological outcomes of breast cancer have received less attention, and such negative outcomes are quite common. Some estimates suggest that 85% of breast cancer survivors suffer from PTSD symptoms (Bulotienė and Matuizienë, 2014) and up to 25% are depressed (National Cancer Institute, 2013). Many breast cancer patients engage in negative cognitive processes such as depressive rumination that includes negative and intrusive thoughts (Steiner et al., 2014). Despite these problems, some studies suggest that only a minority of women who experience some type of meaningful psychological distress following a diagnosis of breast cancer receive some type of treatment for this distress (e.g., Ramachandra et al., 2009).

The present paper aims to examine the influence of a gratitude diary on improving the daily psychological functioning of women treated for breast cancer. Over 2 weeks, half of the participants reported the reasons why they were grateful each day, and the other half did not. Participants also provided measures of their daily psychological functioning (broadly defined) each day. Our general expectation was that listing the reasons why they felt grateful each day would increase daily psychological functioning for our participants. We chose a gratitude intervention (rather than another type of intervention) based on previous research in other populations (Emmons and McCullough, 2003). Moreover, some (Schmidt et al., 2011) have suggested that health psychologists have not examined the potential benefits of gratitude despite the fact that numerous studies have suggested that experiencing gratitude is linked to well-being specifically among populations at risk (e.g., Ruini and Vescovelli, 2013).

### Understanding Gratitude

The intuitive understanding of the word “gratitude” is associated with a thankful response to a gift or someone’s positive action toward us (Lambert et al., 2009). However, gratitude cannot be confused with reciprocity, an internalized obligation to pay someone back with a similar positive action (Watkins et al., 2006).

Gratitude may be defined as an emotion – a temporal state of being grateful for various things in life, such as the presence of beloved people (Lambert et al., 2009). It is also an appraisal and appreciation of altruistic acts toward us (Emmons, 2004). This perspective is the closest to the colloquial understanding of the word. Nevertheless, various researchers (especially Emmons, 2004) have studied gratitude as more than just a fleeting emotion.

Gratitude has been considered a trait, a disposition, or a “life orientation towards noticing and appreciating the positive in the world” (Wood et al., 2010, p. 891). This life orientation is not synonymous with optimism or hope, since, unlike them, it focuses on the present moment (Wood et al., 2010). A person with a high level of dispositional gratitude is able to appreciate others, feel awe when facing beauty and generally, seize the day because of their understanding that life is short (Wood et al., 2010).

The Broaden and Build theory states that gratitude, like other positive emotions, is able to extend people’s range of thoughts and actions and expand their psychological and social resources (Fredrickson, 2004). Gratitude builds enduring resources (e.g., skills for showing appreciation, social bonds), that function as reserves that can be used in difficult times (Fredrickson, 2004, p. 152). When a person is faced with negative situations that may narrow the range of potential actions and thoughts, gratitude may facilitate coping with negative emotions and restoring cognitive flexibility (Fredrickson and Levenson, 1998).

From an evolutionary perspective, positive emotions can be “undoers” of physiological arousal after the experience of negative emotions (Levenson, 1988), and what is more, they build an individual’s resources for survival (Fredrickson, 2013, p. 15). Having experienced something negative, a person relies on psychological resources (e.g., resilience, the ability to recover back from negative events) or social resources (e.g., support from social connections) to return to a positive state (Fredrickson, 2013). Tugade and Fredrickson (2004; Study 1) suggested that highly resilient people use “the undo effect of positive emotions” to help them cope with negative emotions. Gratitude, which is listed at the second place among most frequently felt positive emotions (Fredrickson, 2013, p. 15), also may fulfill the “undoing” function and serve as a resource for regulating consequences of difficult life events.

### Gratitude Interventions

The possibility that people’s daily psychological functioning can be increased by increasing their feelings of gratitude was first examined by Emmons and McCullough (2003) in three studies which varied in the intensity of the intervention, measures and samples. In their first study, participants kept weekly diaries for nine weeks. In the diaries they described events according to the group they were assigned to – the first group described their hassles, the second one – reasons for gratitude, the third one – ordinary daily events. Apart from the diaries, all participants also reported their weekly well-being. The results showed that the gratitude group had significantly higher well-being than the other two groups, which suggests that gratitude may be more effective in promoting well-being than the other types of daily appraisals. This project formed the basis for research into what has come to be called “gratitude interventions.” In a gratitude intervention, participants do something (usually on a regular basis for an extended period of time) that is intended to increase their awareness of the things for which they should feel grateful. A prime example of this is the intervention we used – listing the things for which one is grateful each day for a few weeks.

Although individual studies have found positive effects of gratitude interventions, overall, support for the effectiveness of gratitude interventions is mixed. In a review of research on gratitude, Wood et al. (2010) concluded that “Indeed we believe that the portrayal of gratitude interventions as a key success of positive psychology is somewhat premature, and are alarmed that the effectiveness of these interventions now seems to be taken for granted amongst the positive psychology community …” (p. 898). A similar conclusion was reached in a meta-analysis of gratitude interventions: “Our results provide weak evidence for the efficacy of gratitude interventions” (Davis et al., 2016, p. 25).

Despite these concerns, both Wood et al. and Davis et al. were optimistic about the effects of gratitude interventions and thought that more research was necessary, and it was this optimism and the need for further research that motivated the present study. In addition, Wood et al. (2010) suggested that gratitude interventions may have a bigger impact on people experiencing less positive affect than people experiencing more positive affect. As mentioned previously, many breast cancer patients are known to suffer psychological distress that is related to positive affect, and we believed this made a gratitude intervention particularly appropriate for breast cancer patients.

### Gratitude Among Breast Cancer Patients

We are aware of three studies that have verified relationships between gratitude expressed by breast cancer patients and their well-being, two were correlational (Algoe and Stanton, 2012; Ruini and Vescovelli, 2013) and one was experimental (Otto et al., 2016). Each of the three studies suggests that gratitude interventions may help women who have breast cancer. Otto et al. (2016) found that a gratitude intervention (writing letters expressing gratitude to friends) led to decreased fear of cancer recurrence. Mediational analyses found that this decrease was mediated by individual differences in meaningful goal pursuit.

Ruini and Vescovelli (2013) examined relationships among gratitude, psychological well-being, and distress in women with breast cancer. In their study, participants suffering from breast cancer provided measures of dispositional gratitude, post-traumatic growth, well-being scale, and somatic symptoms. Their results suggested that dispositional gratitude was positively related to all dimensions of a posttraumatic growth scale of well-being and was positively related to relaxation and contentment. In terms of negative outcomes, gratitude was negatively correlated with anxiety, depression, and hostility.

Algoe and Stanton (2012) focused on the social functions of gratitude with a specific emphasis on social support. Women who had been diagnosed with breast cancer listed the people with whom they interacted regularly. Next, they imagined a positive thing that one of these people had done for them recently. After writing a short story, each of the women answered questions regarding the thoughts they had at the moment. They described the emotions they felt, and the intentions they assumed the other people had. Compared to women who felt less gratitude, women who felt more gratitude experienced more social support even 3 months after the end of the study. Social support itself appears to be of paramount importance in the process of healing, and it has been suggested to be related to psychological distress in the breast cancer population (Moyer and Salovey, 1999).

### Within-Person Relationships Between Gratitude and Daily Functioning

Apart from examining the effects of the intervention, we aimed to examine within-person relationships between gratitude and daily psychological functioning. Examining within-person relationships provides an important complement to research on between-person relationships. First, examining within-person relationships involving gratitude is consistent with calls for examining within-person variability in constructs that have been traditionally studied as traits (e.g., Cervone, 2005). Moreover, within- and between-person relationships between the same constructs may present different psychological mechanisms (Affleck et al., 1999), and so one cannot assume that the same relationships exist at the two levels of analysis.

There is an increasing number of studies demonstrating the link between gratitude and daily psychological functioning at the within-person level. For example, Krejtz et al. (2016), Nezlek et al. (2017), and Kashdan et al. (2006), all found that daily gratitude was related to daily well-being. In each of these studies participants maintained a diary comparable to our diary, and the present study was designed (in part) to extend our understanding of such within-person relationships.

### The Present Study

The main objective of the study was to determine if a gratitude intervention, similar to the one introduced by Emmons and McCullough (2003), could improve the daily psychological functioning of oncological patients. Women who took part in the experiment had been diagnosed with, and had been treated for, breast cancer. On the first day of the study, trait measures (such as anxiety, acceptance of illness, coping, well-being) were taken in order to control the dispositional differences between participants. Each day for 2 weeks they provided measures of their daily psychological functioning and related constructs including social support and how they coped with their problems each day. Most of them provided answers online, while some used the paper–pencil method due to their lack of computer skills or internet access.

Daily functioning was defined in terms of self-esteem, optimism, acceptance of illness, and affect. One group of participants kept a diary that started with a list of reasons for gratitude each of the 14 days; the other group kept a diary without listing these reasons. At the between-person level, we expected that women who listed the things for which they were grateful would have better daily functioning, perceive that they had more social support, and cope better than women who did not. At the within-person level, similar to other studies (e.g., Ruini and Vescovelli, 2013), we predicted that daily gratitude would be positively correlated with daily psychological functioning, social support, and coping. Given the lack of existing research and theory about possible effects of a gratitude intervention on relationships at the within-person level, we examined differences between conditions in these relationships on an exploratory basis. We expected that gratitude would affect daily functioning because gratitude, similar to other positive emotions, is known to extend the range of thoughts and actions, and build people’s psychological and social resources (Fredrickson, 2004). The broadening-and-building effect of positive emotions has been previously studied by Han et al. (2008) in breast cancer population. Han et al. (2008) analyzed the content of messages posted on online support groups for women with breast cancer. Interestingly, over a half of the emotional content in analyzed messages was positive, even for health-related stressful situations. It may imply that cancer patients seek positive emotions intuitively, as a potential coping strategy.

## Materials and Methods

### Participants and Procedure

Women with a history of breast cancer were contacted via an oncological foundation and support groups in Poland. They were told that the study would give them an opportunity to spend 15 min every evening with their own thoughts and emotions, allowing them to focus on themselves.

The initial sample was 61, 6 of whom decided not to participate after hearing the instructions for the daily diary and 13 of whom were excluded from the analyses because they did not comply with instructions for maintaining the diary. All participants provided written informed consent confirming that they were free to leave the experiment at any time. To create equivalent groups, we used a table with random numbers to assign participants to condition. The entire procedure was double-blind. The study was approved by the institutional review board for research involving human subjects.

The final sample consisted of 42 women who had been randomly assigned to either the gratitude intervention group (*n* = 21, Mean age = 46.86, *SD* = 9.04) or the control group (*n* = 21, Mean age = 51.62, *SD* = 15.53). The groups did not significantly differ in terms of age [*t* (32) = 1.22, *p* = 0.23], marital status [$\chi {}^{2}$ (3) = 6.8, *p* = 0.08], or whether they were living alone or with someone else during the time of the study [$\chi {}^{2}$ (4) = 7.24, *p* = 0.12]. On average, women in both groups had one child.

We measured stage of treatment, and 38% of women had finished treatment before participating in the study. Seventy-four had received chemotherapy, 55% had received radiotherapy 67% had undergone surgical mastectomy, and a vast majority, 95%, had had some type of operation. The only significant difference between the groups was the time since they found out about cancer. The control group found out about cancer more recently (19 months before the study) than the gratitude group (38 months before the study), *t* (25) = 2.16, *p* = 0.04.

At introductory sessions the goals of the study were explained. Both the experimental and the control group were invited to take part in a project on daily well-being. Participants were told that the study was about different aspects of functioning and everyday life with breast cancer and they were instructed on how to log onto the online platform to record their data. None of the participants were aware that they were taking part in any kind of intervention. Both groups kept diaries for 2 weeks in which they reported on daily events and daily well-being. The only difference between these diaries was the first question about reasons for gratitude (see section “Gratitude Manipulation”), which was presented only to the experimental group. The control group controlled for the possible effects of keeping a diary *per se*.

All participants were asked to provide data in the evening or the early morning of the next day. A majority of participants (63%) provided their data online, whereas the other participants provided their responses using paper forms. Despite the differences between these two methods, research indicates that such differences in data collection are not related to the results of studies of daily experience (Green et al., 2006; Nezlek, 2012). We examined whether the mode of diary procedure (online vs. paper–pencil) had any impact on daily reports over the effect of the experimental manipulation. There were no significant differences in daily measures of participants who kept the diary in an online or paper–pencil form, except for positive deactive affect (*M* = 3.95 for online, *M* = 4.89 for paper–pencil; *t* = 2.12 *p* = 0.04).

### Trait Level Measures

At the beginning of the study, before they began the diary, participants completed a set of individual difference measures. Participants completed the following scales: the trait subscale of the State-Trait Anxiety Inventory (STAI; Spielberger, 1989), acceptance of illness scale (AIS; Felton and Revenson, 1984), stress coping scale (CISS; Endler and Parker, 1990), psychological well-being scale (PWBS; Ryff and Keyes, 1995), gratitude scale (GQ; McCullough et al., 2002), and the Center for Epidemiological Studies Depression Scale (CES-D; Radloff, 1977). The descriptive statistics for these responses are reported in Table 1. The reliabilities for the scales (Cronbach’s alpha) were acceptable. The groups did not significantly differ on the trait level of tested variables before the implementation of the diary procedure (see Table 1).

**TABLE 1:** Descriptive statistics for pre-test measures by group and results of *t*-tests comparing means for control and intervention groups.

**Measure**	**Reliability**	***M* (*SD)***		***t*-ratio**
		**Control**	**Intervention**	
Anxiety (STAI trait)	0.93	2.27 (0.47)	2.16 (0.59)	<1
Acceptance of illness (AIS)	0.86	2.41 (0.84)	2.52 (1.21)	<1
Stress coping – emotional style (CISS)	0.87	2.46 (0.80)	2.67 (0.90)	<1
Stress coping – avoidance style (CISS)	0.87	2.91 (0.45)	2.94 (0.35)	<1
Stress coping – task oriented style (CISS)	0.87	3.56 (0.52)	3.78 (0.49)	1.38
Well-being (PWBS)	0.85	3.39 (0.38)	3.61 (0.66)	1.31
Gratitude (GQ)	0.79	5.07 (0.86)	5.56 (1.31)	1.41
Depression (CES-D)	0.94	0.83 (0.58)	0.75 (0.76)	<1

### Gratitude Manipulation

Before answering questions about daily functioning, the women who were assigned to the gratitude condition reported the reasons for their daily gratitude. They were given the same instructions used by Emmons and McCullough (2003): “There are many things in our lives, both large and small, that we might be grateful about. Think back over the day and write down on the lines below all that you are grateful for today.” They could report up to six reasons.

### Daily Measures

The daily measures were items chosen from existing trait-level scales, adapted for daily administration. Some of the items were selected from the ones that had been used in the past (Krejtz et al., 2016). The procedure of item adaptation followed the guidelines discussed in Nezlek (2012, pp. 29–33).

All participants answered a series of questions about their daily functioning. One set of measures concerned daily affect, daily self-esteem, daily acceptance of illness, and daily optimism, a second set concerned support and coping, and a third set concerned gratitude. Regardless, all daily measures clearly referred to how people felt or thought that day. Items were translated and backtranslated from Polish to English by a research team that included individuals fluent in both languages.

Our measure of daily affect was based on the circumplex model of Feldman Barrett and Russell (1998). This model consists of four separate groups of emotions: positive-active (happy and excited/enthusiastic), positive-deactive (calm, satisfied, and relaxed), negative-active (nervous and angry), negative-deactive (sad, bored, and disappointed). Starting with the words “Today, I felt…,” the women rated emotional adjectives on a 7-point scale (anchored 1 = “Did not feel this way at all” and 7 = “Felt this way very strongly”).

Daily self-esteem was measured with five statements based on the Rosenberg Self-esteem scale (Rosenberg, 1965). Each started with the stem “Today…” The items were: I felt useless, I generally felt satisfied with myself, I felt I had several good traits, I felt that there weren’t many things I could be proud of, and I had a positive attitude toward myself. Participants provided answers ranging from 1 (disagree) to 7 (agree).

Daily acceptance of illness was measured using three items taken from Felton and Revenson (1984). Each item began with the stem “Thinking about today, to what extent would you agree that ….” The items were: because of my physical state, today I’ve been unable to do what I like the most, My health has made me feel less than a full-quality person today, and today I haven’t been self-sufficient to the extent I would like to be. Participants provided answers on a scale anchored 1 (strongly agree), and 5 (strongly disagree). Note that these items refer to the negative effects of illness. To create a positively valent measure that was consistent with the scale label of acceptance, the response scales for the items were reversed so that higher numbers indicated better outcomes.

Daily optimism was composed of four items based on the LOT-R (Scheier et al., 1994). The items started with the following statement: Thinking about today, to what extent would you agree with the following statements. The items were: today, I believed that if a failure was meant to happen to me, it would; today, I had not expected things to turn out the way I wanted; today, I rarely expected something good to happen to me; in general, today I expected to experience more of the good things than the bad ones. Participants provided answers ranging from 1 (disagree) to 7 (agree).

Daily coping was measured with seven items based on Endler and Parker (1990). The items contained both adaptive and non-adaptive coping behaviors. The items were introduced by the following statement: Thinking about today, how did you cope with stressful situations? The items were: I tried to be with other people, I was focused on my physical symptoms, I told myself that it wasn’t real, I was anxious that I wouldn’t cope with it, I thought about the times when I felt better, I regretted I couldn’t change what had happened or what I felt as a consequence, and I approached the problem from different perspectives. Participants answered on a 5-point scale, anchored 1 = never and 5 = very often.

Daily perceived social support was measured using five items, three items taken from Sarason et al. (1987) and two items taken from Pasipanodya et al. (2012) and Belcher et al. (2011). The first three questions measured perceived support from other people. The items started with the stem “How many people have you met today on whom you count regarding the following situations.” The items were: taking care of you regardless of what’s happening; comforting you when you’re sad; accepting you totally, with your strong and weak sides? The response scale ranged from 1 (none) to 7 (more than 5). The other two questions measured support received from life partners: At this moment, how much intimacy/connectedness do you feel with your spouse/partner? Have received support from your partner today in a worry, problem, or difficulty? Participants provided answers ranging from 1 (not at all) to 7 (very much).

Daily gratitude was composed of three questions taken from a scale proposed by McCullough et al. (2002). The items were introduced by the following statement: Thinking about today in general, to what extent do you agree that. The items were: Today, in my life, there were many things that I can be grateful for, If I were to make a list of things I’m grateful for today, it would be a long list, Today it took me a long time to feel grateful for someone or something. Participants provided answers using a scale that ranged from 1 (strongly disagree) to 7 (strongly agree).

We also measured gratitude affectively with the terms “grateful” and “appreciative.” These items were included in the list of affect terms mentioned previously.

### Compliance With Instructions

Prior to the data analyses, we examined participants’ diaries. We excluded from the analyses 13 participants who provided less than 5 days of valid data. The final day-level data consisted of 564 days for 42 participants (*M* = 13.4, *SD* = 2.04, min = 8, max = 19). The data can be accessed on the Open Science Framework^1^.

## Results

The data represent a two-level structure with days nested within participants. A series of multilevel models were carried out in the HLM program (Raudenbush et al., 2011). The analyses were in accordance with the instructions provided by Nezlek (2012).

At the beginning, we examined distributions of our variables and values of skewness and kurtosis were within the acceptable range −2 and +2, not exceeding −1.5 and +1.5 values (George and Mallery, 2010).

### Reliability Analyses of Daily Measures

We started with three level models testing the reliability of the daily measures – the multilevel counterpart of a Cronbach’s alpha. In the models, the items were nested within days and days were nested within individuals (Nezlek, 2017). The reliability estimates for the scales that were used in the analyses are presented in Table 2. In all scales, the higher numbers represented higher values of the measured construct (e.g., higher self-esteem, more optimism). Note that we did not model coping responses as a single scale because we were interested in the individual items.

**TABLE 2:** Descriptive statistics and intervention effects for daily well-being.

		**Means**		**Variances**		**Effect**
**Measure**	**Reliability**	**Control**	**Intervention**	**Between**	**Within**	***t*-ratio**
**Daily gratitude**						
Cognitive	0.73	3.82	5.02	1.64	1.60	3.32^∗∗^
Affective	0.84	4.12	4.92	1.74	1.36	2.04^*^
**Daily functioning**						
Self-esteem	0.68	4.71	5.33	0.73	1.05	2.42^*^
Optimism	0.52	4.68	5.68	1.56	0.98	2.78^∗∗^
Acceptance of illness	0.72	3.42	3.96	0.73	0.76	2.07^*^
**Affect**						
Positive Active	0.55	4.04	4.18	1.21	1.47	<1
Positive Deactive	0.76	4.42	4.20	0.10	1.31	<1
Negative Active	0.84	2.79	2.87	0.92	2.52	<1
Negative Deactive	0.72	2.33	2.29	0.64	1.48	<1
**Social support**						
Partner’s	0.82	2.38	4.26	2.04	1.12	3.35^∗∗^
Other’s	0.84	2.52	3.58	1.30	1.33	3.27^∗∗^
**Coping**						
Being with other people		3.17	3.63	0.67	0.93	$1.83^{a}$
Focus on physical symptoms		2.90	1.81	0.88	0.97	$4.35^{*,**}$
Denial (not real)		2.09	1.47	0.52	0.49	3.04^∗∗^
Anxious could not cope		2.56	2.02	0.73	0.92	2.06^*^
Think about better times		2.78	2.20	0.94	0.83	$1.93^{a}$
Regret what happened		2.67	1.90	0.64	0.98	$3.43^{*,**}$
Different perspective		2.45	3.33	0.62	0.90	$4.22^{*,**}$

^a^*p* < 0.10, ^*^p < 0.05, ^∗∗^*p* < 0.01, ^∗∗∗^*p* < 0.001.

For two of our daily measures these analyses suggested that the items did not constitute reliable scales. For daily cognitive gratitude the reliability was 0.18. Answers to the third question “Today, how much time did it have to go before you felt grateful to someone or something?” were not highly related to answers to the first two questions. As a result, daily gratitude was calculated as a mean of the first two questions.

A similar situation occurred for our measure of daily optimism. The estimated reliability of a scale that used all four items was 0.16. Answers to the fourth item “In general today I’ve expected to experience more of the good things than the bad ones” were not highly related to answers to the first three questions. As a result, daily optimism was calculated as a mean of the first three questions.

It is important to note that the reliabilities of scales in diary studies, tend to be lower than the reliabilities of the corresponding trait level measures (Nezlek, 2017). This can be due to the fact that scales adapted for daily administration usually have fewer items than trait level measures of the same constructs. Holding the inter-item correlations constant, alpha decreases as the number of items decreases (Carmines and Zeller, 1979, p. 46). Nevertheless, the MLM analyses we used incorporated “Bayes shrinkage.” Bayes shrinkage refers to the fact that MLM analyses take into account the reliability of coefficients when estimating variances, covariances, and significance tests (Raudenbush and Bryk, 2002).

### Descriptive Statistics of Daily Measures

The first analyses were null models (no predictors at either level of analysis), which estimated the basic multilevel descriptive statistics, the mean, and the between- and within-person variance estimates. The basic model is presented below. In terms of nomenclature, there were i days nested within j persons, β represents the mean (for j persons), the variance of *r*_ij_ represents the level-1 (within-person) variance, $\gamma {}_{00}$ represents the grand mean (the mean of β for each person), and the variance of *u*_0j_ represents the level-2 (between-person) variance. The results of these analyses are presented in Table 2.

$$\mathrm{Within}-\mathrm{person}:y_{\mathrm{ij}}={\text{β}}_{0\,j}+r_{\mathrm{ij}}$$

$$\mathrm{Between}-\mathrm{person}:{\text{β}}_{0\,j}=\gamma_{00}+u_{0\,j}$$

### Effect of Gratitude Manipulation on Daily Functioning

To examine the effect of the gratitude intervention on daily functioning, we added a condition variable at the between-person level to the equation shown above (coded 1 = intervention, −1 = control). Table 2 summarizes the findings of these models, including estimated means and between- and within-person variance for the two conditions. The results (t-ratio) of the significance test of the condition effect are in the column labeled “Effect.”

As can be seen from Table 2, the gratitude intervention led to increases in daily gratitude in terms of both affective and cognitive measures of gratitude. The intervention group reported significantly higher daily cognitive gratitude (*M* = 5.02) and affective gratitude (*M* = 4.92), compared to the control group (*M* = 3.82, 4.12, respectively, *p* < 0.05). Consistent with our expectations, women in the intervention condition reported higher daily self-esteem (*M* = 5.33), optimism (*M* = 5.68), and acceptance of illness (*M* = 3.96), than women in the control group (*M* = 4.71, 4.68, 3.42, respectively, *p*s < 0.05). In contrast, there were no significant differences in measures of affect. We found similar differences in our analyses of social support and coping mechanisms. Women in the gratitude intervention condition perceived more social support from their partners (*M* = 4.26) and from others in general (*M* = 3.58), comparing to the control group (*M* = 2.38, 2.52, respectively, *p* < 0.01). Women in the intervention condition also reported using more adaptive coping mechanisms (e.g., taking a different perspective, *M* = 3.33) more often than women in the control condition (*M* = 2.45, *p* < 0.001), and they reported using less adaptive coping mechanisms (e.g., denial; *M* = 1.47) less often than women in the control condition (*M* = 2.09, *p* < 0.01). Although statistical comparisons of individual items were not possible within the multilevel framework we used, the mean differences suggested that the intervention may have been more effective at reducing maladaptive coping than it was at increasing the use of adaptive coping.

On an exploratory basis we examined relationships between time of diagnosis and our daily measures. Two women were excluded from these analyses because their time since diagnosis was more than 3 *SD* from the mean for the sample, 28.48 (29.32) months. These analyses found no significant relationships between time since diagnosis and our daily outcome measures.

### Within-Person Relationships Between Gratitude and Daily Functioning

In the third step of the analyses, we tested the within-person relationships between daily gratitude and other aspects of daily functioning. We had no specific predictions about whether these relations would differ depending on how gratitude was measured, and so we analyzed both measures of gratitude. We should note that the estimated within-person correlation between the two aspects of gratitude (cognitive and affective) was 0.65, suggesting meaningful overlap in the two constructs. For both measures, daily gratitude was entered group-mean centered and was modeled as randomly varying. The model is below, and Table 3 provides details of these models.

**TABLE 3:** Within-person relationships between affective and cognitive gratitude and daily well-being.

**Outcome measure**	**Affective**		**Cognitive**	
	**Coefficient**	***t*-ratio**	**Coefficient**	***t*-ratio**
Self-esteem	0.53	$9.94^{*,**}$	0.40	$9.53^{*,**}$
Optimism	0.17	3.28^∗∗^	0.15	3.65^∗∗^
Acceptance of illness	0.23	$5.33^{*,**}$	0.18	$4.73^{*,**}$
**Affect**				
Positive Active	0.71	$12.28^{*,**}$	0.52	$9.95^{*,**}$
Positive Deactive	0.64	$13.93^{*,**}$	0.38	$7.23^{*,**}$
Negative Active	–0.51	$4.41^{*,**}$	–0.12	1.07
Negative Deactive	–0.56	$7.92^{*,**}$	–0.29	$4.35^{*,**}$
**Social support**				
Partner’s	0.24	$3.86^{*,**}$	0.15	2.96^∗∗^
Others’	0.19	3.09^∗∗^	0.32	$5.96^{*,**}$
**Coping**				
Being with other people	0.23	$5.33^{*,**}$	0.27	$6.82^{*,**}$
Focus on physical symptoms	–0.18	2.70^∗∗^	–0.07	1.57
Denial (not real)	–0.05	1.31	–0.01	<1
Anxious could not cope	–0.07	1.15	–0.14	2.90^∗∗^
Think about better times	–0.22	$4.88^{*,**}$	–0.11	3.41^∗∗^
Regret what happened	–0.21	$4.29^{*,**}$	–0.14	3.12^∗∗^
Different perspective	0.05	<1	0.08	$1.73^{a}$

*^a^*p* < 0.10, ^∗∗^*p* < 0.01, ^∗∗∗^*p* < 0.001.*

$$\mathrm{Within}-\mathrm{person}:y_{\mathrm{ij}}={\text{β}}_{0\,j}+{\text{β}}_{1\,j}\times \left(\mathrm{Daily}\,\mathrm{gratitude}\right)+r_{\mathrm{ij}}$$

$$\mathrm{Between}-\mathrm{person}:{\text{β}}_{0\,j}=\gamma_{00}+u_{0\,j}$$

$${\text{β}}_{1\,j}=\gamma_{10}+u_{1\,j}$$

In line with our expectations, we found that both gratitude measures (cognitive and affective) were positively correlated with positive aspects of daily functioning (e.g., positive active affect and self-esteem), and were negatively correlated with negative aspects of well-being (negative active and deactive affect), with the exception of negative active affect and the cognitively focused measure of gratitude. Women’s daily psychological functioning was found to be better when they experienced more gratitude, compared to their functioning on days when the felt less grateful. We should note that group-mean centering controlled for individual differences in means of the daily gratitude measures.

As expected, and as summarized in Table 3, we found that both gratitude measures were positively related to perceived support from partners and others. In addition, daily gratitude was negatively related to the use of less adaptive coping behaviors (e.g., regret) and was positively related to more adaptive coping behavior, being with other people. Interestingly, daily gratitude was not significantly related to the adaptive coping mechanism of taking a different perspective, whereas there was a significant effect for the intervention at the between-person level of analysis of this measure.

Finally, we conducted analyses to determine if the intervention moderated within-person relationships between daily gratitude and the other aspects of daily functioning. We did this by adding a contrast-coded predictor to both equations of the model that was used to examine relationships between daily gratitude and the other aspects of daily functioning. These analyses found only isolated moderating effects indicating that relationships between gratitude and other daily measures did not vary consistently between the intervention and control conditions.

## Discussion

The primary focus of the present paper was to determine if a gratitude intervention could improve the daily functioning of women with breast cancer. Our results clearly indicated that this is possible. The women in our study who wrote down the reasons why they felt grateful every day were found to have better daily functioning in terms of numerous measures compared to women who did not do this. A secondary focus of the study was to replicate previous research that has found within-person relationships between daily functioning and daily gratitude (Krejtz et al., 2016; Nezlek et al., 2017). We replicated these relationships, but did not find that they varied between the intervention and control conditions.

### Gratitude Interventions for Women With Breast Cancer

Our results complement and extend existing research on gratitude interventions *per se* and on how these interventions function in women with breast cancer, a sample clearly at-risk for psychological distress. We are aware of only one experimental study, Otto et al. (2016) that has examined the effects of a gratitude intervention on women with breast cancer. Otto et al. found that their intervention increased positive affect and reduced a specific construct, fear of death due to the recurrence of cancer, although the intervention had no effect on general fear of death. Otto et al. did not report collecting any other measures, and so our results for self-esteem, optimism, accepting illness, social support, and coping meaningfully extend the domain of the possible benefits of a gratitude intervention with this population.

We believe that it is important to determine the effects gratitude interventions can have on women with breast cancer. It cannot be assumed that the same effects will occur for women with breast cancer that have been found for gratitude interventions for people who are not facing the same challenges these women face (e.g., Krejtz et al., 2016). Women with breast cancer face different life challenges than healthy individuals or individuals suffering from other types of cancer. Challenges that might be unique or more pronounced for breast cancer patients than they are for others could be rooted in the areas of self-esteem issues, such as body image, sexuality, intimacy and sense of femininity (Miller et al., 2016). All patients in the present study had undergone mastectomy, which could be an important source of the above-mentioned difficulties; as Odgen and Lindridge (2008) suggested breast scarring has a tremendous negative effect on not only on the patients’ perception of self, but also on other people’s judgment of their attractiveness and self-esteem.

We were concerned that these challenges would render ineffectual the types of interventions that have worked with the general population. Fortunately, it appears that these challenges do not render gratitude interventions powerless.

Along the same lines, as an intervention based on a positive psychology model, the present study complements and extends the existing research on how positive psychological principles can be applied to helping people suffering from breast cancer. Casellas-Grau et al. (2014) concluded that although different types of interventions based on positive psychology appeared to improve the well-being of breast cancer patients, the quality of the existing research was such that firm conclusions about effectiveness were not possible. Moreover, and understandably given the lack of research when they conducted their review, gratitude interventions were not mentioned in their review. Through the use of random assignment to control and treatment conditions and the use of sophisticated sampling and data analysis techniques, we believe that the present study addresses many of the shortcomings Casellas-Grau et al. (2014) described.

In terms of gratitude interventions *per se*, in addition to expanding the domain of possible benefits of these interventions, we also found that our intervention increased how grateful participants felt. This is at odds with the results of Davis et al. (2016). In their meta-analysis they concluded that levels of gratitude were not influenced by gratitude interventions vs. measurement-only controls. The significant difference in the present study may simply reflect random variability. For example, using similar methods and measures with a healthy population, Krejtz et al. (2016) did not find that their gratitude intervention affected levels of gratitude, although the difference was in the expected direction at *p* < 0.10.

The other possibility is that intervention was more powerful for women with breast cancer than it would have been with a sample that was less distressed. Such a difference is suggested by a comparison of the mean scores for gratitude in the control and intervention samples in the present study (3.82 and 5.02, respectively) with mean scores for gratitude for the control and intervention samples presented in Krejtz et al. (2016; M = 4.31 and M = 4.67, respectively). Although statistical tests are not possible, the present control sample felt less grateful than participants in Krejtz et al. (2016). Given random assignment, we can assume that the women who were in the present intervention condition would have reported similar levels of gratitude to women in the control condition if the intervention had not occurred.

It is easy to understand why women with breast cancer might have lower levels of gratitude than the general population. They have experienced a major, life-altering trauma. If gratitude consists of thinking about the good things in life, women with breast cancer are reminded every day about a very important bad thing in their lives, which may make it more difficult to think of the good things. Such a possibility could explain why the gratitude intervention had such a strong effect on these women (over a scale point). Reminding them each day of the good things in their lives for which they could feel grateful probably contrasted strongly with their normal everyday thinking, more strongly than it might for the general population.

### How Gratitude Works: A Potential Mechanism Explaining the Effectiveness of Gratitude Intervention

Although our study was not designed to examine the mechanisms that might underlie relationships between gratitude and functioning, these mechanisms might be explained by the Broaden and Build Theory (Fredrickson, 1998, 2001). It suggests that positive emotions, including gratitude, “undo” the consequences of negative events. It is possible that gratitude is an “undoer” of distress related to breast cancer treatment. Perhaps our gratitude intervention worked because there is room for recovery among women with breast cancer.

There are individual differences in how much individuals seek positive emotions in their daily lives (Catalino et al., 2014). “Prioritizing positivity” is how they called the tendency “to seek out positivity by virtue of how they make decisions about how to organize their day-to-day lives” (Catalino et al., 2014, p. 1159). Catalino et al. (2014) suggest that individuals who prioritize positivity in everyday life by selecting situations that maximize their positive emotions tend to feel happier (Catalino et al., 2014). Interpreting daily gratitude as a strategy of prioritizing positivity serves a convincing scenario, suggesting that active expressing gratitude might be an effective strategy to increase daily functioning.

### Within-Person Relationships Between Gratitude and Well-Being

The other focus of the paper was to verify within-person relationships between gratitude and well-being. Consistent with the literature (Kashdan et al., 2006; Krejtz et al., 2016; Nezlek et al., 2017) we found that daily gratitude was positively correlated with positive aspects of daily functioning. Our results replicate the results of previous research that studied veterans of the war in Vietnam (Kashdan et al., 2006), healthy adults (Krejtz et al., 2016), and college students (Nezlek et al., 2017), suggesting that these relations are not sample-specific. Moreover, some of the outcome measures of the present study were different than those used previously, suggesting that the effects of daily gratitude are broad. Establishing the universality of within-person relationships between gratitude and daily psychological functioning will require work involving different samples and different measures. Nevertheless, we think the present study in combination with existing research provides a good starting point.

### eHealth Interventions Versus Non-digital Interventions

In the present study, the majority of the participants kept an online diary, whereas 37% kept its non-digital counterpart. Although the method effect was not significant for any of the daily measures for which the condition effect was significant, it may be worth conducting a study in which the method effect would be examined in two equal-sized groups, as the advantages of digital interventions are largely discussed nowadays. There is a substantial body of research suggesting that eHealth interventions are effective at improving the well-being of women with breast cancer (Triberti et al., 2019). eHealth diaries maintain participants’ engagement because they are interactive and accessible at any time. Nevertheless, Triberti et al. (2019) claim that the effectiveness of eHealth interventions largely depends on patients’ attitude toward the potential effectiveness of such much and we are unaware of research on this specific topic. Moreover, eHealth interventions are often expected to be more effective than they are in reality (Granja et al., 2018). This difference may be due to various factors such as disrupted workflow and a loss of face-to-face communication reported by doctors whose patients started using digital interventions.

Børøsund et al. (2014) studied the effect of several Web-based health programs on the functioning of breast cancer patients and compared it to the traditional care outcomes. The results indicated significantly lower anxiety and depression in the digital intervention groups (compared to the non-digital intervention group). The authors attributed some of these differences to the fact that the online interventions were multi-componential and provided more functions than traditional care programs (Børøsund et al., 2014). Online interventions have a natural advantage of enabling communication, interaction, and support. Nevertheless, as long as the content and functionalities of digital and non-digital interventions are the same, it may be expected that they would not differ in their outcome simply because of the mode of application (Triberti et al., 2019). In the present study, the online diary and the paper–pencil one had the same content, which may explain the lack of the method effect.

### Limitations and Future Directions

Although we believe the present study furthers our understanding of the gratitude intervention as a contribution to the literature, the study is not free from shortcomings. Even though we do not suspect that the present sample differs systematically from the population of women with breast cancer, the present sample may not be representative of women with breast cancer. The sample size was not large, and it is possible that a larger sample may have found differences that we did not (e.g., a moderating effect of the intervention on within-person relationships between gratitude and daily functioning). Moreover, future research might compare the possible differences between different types of cancer, including cancers that are limited to men (e.g., prostate cancers).

In the present study, although the assignment was random, the groups differed in terms of time since original diagnosis. No significant relationships were found between time since diagnosis and daily functioning. Nevertheless, future studies should control for survival time, possible treatment complications, and overall baseline functioning of cancer patients. There are many kinds of treatments. For example, some types of breast cancer require hormonal treatment that lasts 10 years after chemotherapy is completed (Miller et al., 2016). Each case is different and probably a more important issue would be to control the character of treatment and other co-existing conditions (e.g., if there are metastases).

We believe that future research on gratitude interventions needs to focus clearly on the mechanisms underlying relationships between gratitude and daily functioning. Such research can take the form of investigating mediating variables, such as the study reported by Otto et al. (2016). Another approach to this issue involves comparing the impact that different types of interventions have on daily psychological functioning. For example, does a gratitude intervention produce different outcomes than a “positive event” intervention such as the “three good things in life” technique used by Killen and Macaskill (2014)? Regardless of the approach, we believe research about these explanatory mechanisms is necessary. From a practical standpoint, interventions may be improved if we have a better understanding of how they work. Moreover, we believe that a follow-up measurement of the patients’ physical condition is worth pursuing in the future.

At the least, the present study suggests that gratitude interventions can be an effective autotherapy for women with breast cancer. Aside from some nominal costs associated with organizing the materials, the intervention we used was inexpensive and did not require staff with advanced degrees. In this sense, we think that it represents an ideal positive psychology intervention. It focuses on building people’s strengths and does so without the overhead (and possible stigma) associated with more traditional forms of therapy.

## Data Availability

All datasets generated for this study are included in the manuscript and/or supplementary files.

## Ethics Statement

This study protocol was approved by the Ethics Committee of SWPS University of Social Sciences and Humanities, Warsaw, Poland. All subjects gave written informed consent in accordance with the Declaration of Helsinki.

## Author Contributions

JS designed and conducted the study, and drafted the manuscript. IK supervised the study. JN provided the conceptual and methodological guidance for all aspects of the project. JS and IK performed the initial data interpretation. IK and JN performed the further advanced data analyses, and edited and reviewed the manuscript. All authors approved the version of the manuscript to be published, and agreed to be accountable for all aspects of the work.

## Conflict of Interest Statement

The authors declare that the research was conducted in the absence of any commercial or financial relationships that could be construed as a potential conflict of interest.

## Footnotes

1 https://osf.io/vpfdr/?view_only=69587cb9a41d4363965e29813b49ea41
